# Supplementary material for: Detecting communicative intent in a computerised test of joint attention
Source: PeerJ. 2017 Jan 17;5:e2899. doi: 10.7717/peerj.2899 (PMC5244879; doi:10.7717/peerj.2899)
Supplement: Supplemental Information 1 [file peerj-05-2899-s001.html]

Supplementary Material 1: R analysis of saccadic reaction times


# Supplementary Material 1: R analysis of saccadic reaction times

```
# Load required packages
library(ez)
library(ggplot2)
library(reshape2)
library(BayesFactor)
library(lme4)
library(lmerTest)

# Read data file and select Respond trials
Data <- read.csv("output/data/TrialData.csv", stringsAsFactors=FALSE)
RespondData <- Data [(Data$SubjectRole=="Respond"), ]

# Identify factors
RespondData$SubNum <- factor(RespondData$SubNum)
RespondData$TrialID <- factor(RespondData$TrialID)
RespondData$Condition <- factor(RespondData$Condition)
RespondData$Task <- factor(RespondData$Task, levels = c("Search","NoSearch")) # Order levels for plots

# Centre factors for lme analysis
RespondData$ConditionC <- ifelse(RespondData$Condition=="RJA",0.5,ifelse(RespondData$Condition=="RJAc",-0.5,NA))
RespondData$TaskC <- ifelse(RespondData$Task=="Search",0.5,ifelse(RespondData$Task=="NoSearch",-0.5,NA))
```

## ANOVA on mean saccadic reaction time

In the paper, we analyse the mean reaction times for correct trials. We trimmed trials with reaction times less than 150ms as these are likely to be anticipations.

```
ScreenedData <- RespondData[which(RespondData$CorrectResponse==1 & RespondData$FirstSaccadeSRT>150),]
BySubjectsData <- aggregate(x = ScreenedData$FirstSaccadeSRT, list(SubNum = ScreenedData$SubNum, Condition = ScreenedData$Condition, Task = ScreenedData$Task), FUN=mean)
ggplot(BySubjectsData, aes(factor(Condition), y=x, fill=Task))+
  geom_boxplot(outlier.colour=NA)+
  scale_fill_brewer(palette="RdBu") + 
  theme_minimal(base_size = 18, base_family = "")+
  scale_x_discrete("")+
  geom_point(position=position_jitterdodge(dodge.width=0.75, jitter.width=0.3), alpha=0.2, size=3)+
  scale_y_continuous("Saccadic Reaction Time / ms",limits=c(0,1000), breaks=seq(0, 1000, 200))
```

ANOVA shows effects of Condition (RJa vs RJAc) and Task (Search vs NoSearch). As predicted, there was also a significant interaction between Condition and Task

```
ezANOVA(BySubjectsData, dv = x, wid = SubNum, within = .(Condition, Task), between = NULL, type = 3)
```

```
## $ANOVA
##           Effect DFn DFd        F            p p<.05       ges
## 2      Condition   1  15 86.33668 1.301783e-07     * 0.5526201
## 3           Task   1  15 10.76380 5.053748e-03     * 0.1205873
## 4 Condition:Task   1  15 47.71347 4.997711e-06     * 0.1622947
```

T-tests show this interaction arises because the effect of Search task is significant for the RJA condition but not for the RJAc condition.

```
t.test(x ~ Task, BySubjectsData[(BySubjectsData$Condition=="RJA"),], paired=TRUE)
```

```
## 
##  Paired t-test
## 
## data:  x by Task
## t = 4.9146, df = 15, p-value = 0.0001869
## alternative hypothesis: true difference in means is not equal to 0
## 95 percent confidence interval:
##   74.3403 188.2055
## sample estimates:
## mean of the differences 
##                131.2729
```

```
t.test(x ~ Task, BySubjectsData[(BySubjectsData$Condition=="RJAc"),], paired=TRUE)
```

```
## 
##  Paired t-test
## 
## data:  x by Task
## t = -0.8729, df = 15, p-value = 0.3965
## alternative hypothesis: true difference in means is not equal to 0
## 95 percent confidence interval:
##  -38.94428  16.31482
## sample estimates:
## mean of the differences 
##               -11.31473
```

We can also look at the effect of Condition across the two tasks. In both Search and NoSearch tasks, responses are slower for RJA than RJAc.

```
t.test(x ~ Condition, BySubjectsData[(BySubjectsData$Task=="Search"),], paired=TRUE)
```

```
## 
##  Paired t-test
## 
## data:  x by Condition
## t = 9.1082, df = 15, p-value = 1.683e-07
## alternative hypothesis: true difference in means is not equal to 0
## 95 percent confidence interval:
##  192.5029 310.1245
## sample estimates:
## mean of the differences 
##                251.3137
```

```
t.test(x ~ Condition, BySubjectsData[(BySubjectsData$Task=="NoSearch"),], paired=TRUE)
```

```
## 
##  Paired t-test
## 
## data:  x by Condition
## t = 7.6413, df = 15, p-value = 1.508e-06
## alternative hypothesis: true difference in means is not equal to 0
## 95 percent confidence interval:
##   78.39822 139.05392
## sample estimates:
## mean of the differences 
##                108.7261
```

## Bayesian analysis on mean saccadic reaction time

We get a complementary pattern of results using Bayesian statistics. There is strong evidence for a Task by Condition interaction.

```
bfMainEffects = lmBF(x ~ Condition + Task, data = BySubjectsData)
bfInteraction = lmBF(x ~ Condition + Task + Condition:Task, data = BySubjectsData)
bf = bfInteraction / bfMainEffects
bf
```

```
## Bayes factor analysis
## --------------
## [1] Condition + Task + Condition:Task : 24.33298 ±4.34%
## 
## Against denominator:
##   x ~ Condition + Task 
## ---
## Bayes factor type: BFlinearModel, JZS
```

Bayesian t-tests show strong evidence for an effect of Task for RJA but evidence favouring the null hypothesis for RJAc.

```
DataWide <- dcast(BySubjectsData, SubNum ~ Condition + Task, value.var="x")
ttestBF(x = DataWide$RJA_Search,y=DataWide$RJA_NoSearch, paired=TRUE)
```

```
## Bayes factor analysis
## --------------
## [1] Alt., r=0.707 : 164.4705 ±0%
## 
## Against denominator:
##   Null, mu = 0 
## ---
## Bayes factor type: BFoneSample, JZS
```

```
ttestBF(x = DataWide$RJAc_Search,y=DataWide$RJAc_NoSearch, paired=TRUE)
```

```
## Bayes factor analysis
## --------------
## [1] Alt., r=0.707 : 0.3557952 ±0.01%
## 
## Against denominator:
##   Null, mu = 0 
## ---
## Bayes factor type: BFoneSample, JZS
```

### Re-analysis of untrimmed data

The predicted interaction remains if we take the mean of the untrimmed data.

```
ScreenedData <- RespondData[which(RespondData$CorrectResponse==1 & RespondData$FirstSaccadeSRT>0),]
BySubjectsData <- aggregate(x = ScreenedData$FirstSaccadeSRT, list(SubNum = ScreenedData$SubNum, Condition = ScreenedData$Condition, Task = ScreenedData$Task), FUN=mean)
ezANOVA(BySubjectsData, dv = x, wid = SubNum, within = .(Condition, Task), between = NULL, type = 3)
```

```
## $ANOVA
##           Effect DFn DFd        F            p p<.05       ges
## 2      Condition   1  15 82.26502 1.776186e-07     * 0.5496822
## 3           Task   1  15 10.42454 5.623367e-03     * 0.1132362
## 4 Condition:Task   1  15 46.93777 5.495974e-06     * 0.1592091
```

The interaction also remains if we take the median rather than the mean reaction time (although the main effect of Task is no longer significant)

```
ScreenedData <- RespondData[which(RespondData$CorrectResponse==1 & RespondData$FirstSaccadeSRT>0),]
BySubjectsData <- aggregate(x = ScreenedData$FirstSaccadeSRT, list(SubNum = ScreenedData$SubNum, Condition = ScreenedData$Condition, Task = ScreenedData$Task), FUN=median)
ezANOVA(BySubjectsData, dv = x, wid = SubNum, within = .(Condition, Task), between = NULL, type = 3)
```

```
## $ANOVA
##           Effect DFn DFd           F            p p<.05         ges
## 2      Condition   1  15 45.99160705 6.181723e-06     * 0.379753198
## 3           Task   1  15  0.09125936 7.667304e-01       0.001028351
## 4 Condition:Task   1  15  5.58304495 3.206668e-02     * 0.032179557
```

### Re-analysis: mixed random effects

We also re-analysed the data taking a mixed random effects approach. To ensure that the residuals were normally distributed, we applied an inverse transformation on reaction times. The success of this transformation is demonstrated by the near-linear quantile-quantile plot. Note that for this transformation to work, we do have to trim reaction times below 150ms.

```
ScreenedData <- RespondData[which(RespondData$CorrectResponse==1 & RespondData$FirstSaccadeSRT>150),]
ScreenedData$invSRT <- -1/ScreenedData$FirstSaccadeSRT
M1 <- lmer(invSRT ~ ConditionC * TaskC + (1|SubNum) + (1|TrialID), ScreenedData)
qqnorm(residuals(M1))
```

Again, we find the predicted interaction between Task and Condition.

```
summary(M1)
```

```
## Linear mixed model fit by REML t-tests use Satterthwaite approximations
##   to degrees of freedom [merModLmerTest]
## Formula: invSRT ~ ConditionC * TaskC + (1 | SubNum) + (1 | TrialID)
##    Data: ScreenedData
## 
## REML criterion at convergence: -37766.2
## 
## Scaled residuals: 
##     Min      1Q  Median      3Q     Max 
## -3.8629 -0.7060 -0.0422  0.6509  3.3265 
## 
## Random effects:
##  Groups   Name        Variance  Std.Dev. 
##  TrialID  (Intercept) 2.735e-08 0.0001654
##  SubNum   (Intercept) 7.355e-08 0.0002712
##  Residual             5.100e-07 0.0007141
## Number of obs: 3263, groups:  TrialID, 216; SubNum, 16
## 
## Fixed effects:
##                    Estimate Std. Error         df t value Pr(>|t|)    
## (Intercept)      -2.730e-03  6.986e-05  1.580e+01 -39.080  < 2e-16 ***
## ConditionC        7.645e-04  3.368e-05  2.132e+02  22.702  < 2e-16 ***
## TaskC             3.930e-05  2.505e-05  3.045e+03   1.569    0.117    
## ConditionC:TaskC  3.241e-04  5.010e-05  3.045e+03   6.471 1.13e-10 ***
## ---
## Signif. codes:  0 '***' 0.001 '**' 0.01 '*' 0.05 '.' 0.1 ' ' 1
## 
## Correlation of Fixed Effects:
##             (Intr) CndtnC TaskC
## ConditionC  0.001              
## TaskC       0.006  0.002       
## CndtnC:TskC 0.000  0.024  0.008
```

Finally, we performed the mixed random effects analysis with a fully-saturated random effects structure (cf. Barr, 2013). In this case, we have random slopes and interactions for each subject as well as random intercepts. Again, we find the predicted Task by Condition interaction.

```
summary(lmer(invSRT ~ ConditionC * TaskC + (1+ConditionC*TaskC|SubNum) + (1|TrialID), ScreenedData))
```

```
## Linear mixed model fit by REML t-tests use Satterthwaite approximations
##   to degrees of freedom [merModLmerTest]
## Formula: 
## invSRT ~ ConditionC * TaskC + (1 + ConditionC * TaskC | SubNum) +  
##     (1 | TrialID)
##    Data: ScreenedData
## 
## REML criterion at convergence: -37976.5
## 
## Scaled residuals: 
##     Min      1Q  Median      3Q     Max 
## -3.3915 -0.6782 -0.0584  0.6111  3.6658 
## 
## Random effects:
##  Groups   Name             Variance  Std.Dev.  Corr             
##  TrialID  (Intercept)      2.493e-08 0.0001579                  
##  SubNum   (Intercept)      7.403e-08 0.0002721                  
##           ConditionC       5.826e-08 0.0002414  0.28            
##           TaskC            1.133e-07 0.0003366  0.02  0.15      
##           ConditionC:TaskC 8.895e-08 0.0002982  0.19  0.53 -0.21
##  Residual                  4.661e-07 0.0006827                  
## Number of obs: 3263, groups:  TrialID, 216; SubNum, 16
## 
## Fixed effects:
##                    Estimate Std. Error         df t value Pr(>|t|)    
## (Intercept)      -2.730e-03  6.990e-05  1.570e+01 -39.053  < 2e-16 ***
## ConditionC        7.632e-04  6.840e-05  1.780e+01  11.158 1.83e-09 ***
## TaskC             4.364e-05  8.751e-05  1.493e+01   0.499  0.62528    
## ConditionC:TaskC  3.180e-04  8.866e-05  1.513e+01   3.586  0.00267 ** 
## ---
## Signif. codes:  0 '***' 0.001 '**' 0.01 '*' 0.05 '.' 0.1 ' ' 1
## 
## Correlation of Fixed Effects:
##             (Intr) CndtnC TaskC 
## ConditionC   0.243              
## TaskC        0.024  0.132       
## CndtnC:TskC  0.153  0.399 -0.166
```

## References

Barr, D. J. (2013). Random effects structure for testing interactions in linear mixed-effects models. Frontiers in Psychology, 4, 328.
