# Supplementary material for: Detecting communicative intent in a computerised test of joint attention
Source: PeerJ. 2017 Jan 17;5:e2899. doi: 10.7717/peerj.2899 (PMC5244879; doi:10.7717/peerj.2899)
Supplement: Supplemental Information 2 [file peerj-05-2899-s002.docx]

**Supplementary Material 2: Subjective task ratings**

**Method.**

After playing the interactive game, participants rated the difficulty, naturalness, intuitiveness, and pleasantness of the social and control conditions on a 5-point Likert scale (1 = not at all, 5 = extremely). They also rated whether they preferred playing the game with Alan or alone on a 10-point scale (0 = complete preference for social interaction, 10 = complete preference for completing the task alone). Participants were also asked to rate how co-operative Alan was on the same scale. This provided an opportunity for participants to declare whether they disbelieved that Alan was a real person. At the end of the study, participants were told that Alan was controlled by a computer program. They were then asked to rate how convinced they were that Alan had been controlled by another human on a 5-point scale (one rating for the whole experiment).

Non-parametric tests (related samples Wilcoxon Signed Ranks Test) were used on all measures of subjective experience since these data violated assumptions of normality. A significance criterion of *p* < 0.05 was used for all analyses.

**Results.**

For both the social and control conditions, there were no significant differences between the Search and NoSearch tasks on ratings of task difficulty, naturalness, intuitiveness, pleasantness, or cooperativeness (all *p*s > .066). Summary statistics are reported in Table 1.

**Belief that the avatar was controlled by a real person*.*** All participants said that they were convinced that the avatar was controlled by a real person, and rated the strength of this belief as either a 4/5 or 5/5 (*median = 5*). The five participants who provided a 4/5 rating, commented that they considered the possibility that Alan could be computer-controlled, but disregarded it. Participants’ beliefs about whether they are engaged in a genuine social interaction may be important, and have been found to influence the neural processing of an avatar’s gaze behaviour in the context of joint attention interactions (Caruana, de Lissa et al., 2016).

**Preference for social condition over control condition*.*** On average, participants reported preferences for playing the interactive task (i.e., social condition), rather than on their own (i.e., control condition). This preference was statistically equivalent for both the Search (*median = 3*) and NoSearch tasks (*median = 3.5*; *V* = 40.5, *p* = .750).

| **Table 1.** *Summary statistics for subjective task ratings by condition and task.* | | | | | |
| --- | --- | --- | --- | --- | --- |
|  | **Task Version** | |  | **Statistics** | |
|  | **Search** | **NoSearch** |  | ***V*** | ***p*** |
| **Social Condition** |  |  |  |  |  |
| Difficulty | 2.0 | 1.0 |  | 38 | .066 |
| Naturalness | 4.0 | 4.0 |  | 16 | .777 |
| Intuitiveness | 4.0 | 4.5 |  | 16 | .243 |
| Pleasantness | 4.0 | 3.0 |  | 12 | .824 |
| Cooperativeness | 5.0 | 5.0 |  | 9 | .766 |
| **Control Condition** |  |  |  |  |  |
| Difficulty | 1.0 | 1.0 |  | 18.5 | .106 |
| Naturalness | 4.0 | 4.0 |  | 20 | .803 |
| Intuitiveness | 4.5 | 5.0 |  | 3 | .066 |
| Pleasantness | 3.0 | 3.5 |  | 10.5 | .588 |
| *Note.* Experience ratings were provided on a 5-point scale (1 = not at all, 5 = extremely). Task effects were tested using a two-sample Wilcoxon signed rank test with continuity correction. | | | | | |
